# *miR-205-5p*-mediated downregulation of ErbB/HER receptors in breast cancer stem cells results in targeted therapy resistance

**DOI:** 10.1038/cddis.2015.192

**Published:** 2015-07-16

**Authors:** A De Cola, S Volpe, M C Budani, M Ferracin, R Lattanzio, A Turdo, D D'Agostino, E Capone, G Stassi, M Todaro, C Di Ilio, G Sala, M Piantelli, M Negrini, A Veronese, V De Laurenzi

**Affiliations:** 1 Department of Experimental and Clinical Sciences, Aging Research Center (Ce.S.I.), University G. D'Annunzio, Chieti-Pescara, Italy; 2 Department of Surgical and Oncological Sciences, University of Palermo, Palermo, Italy; 3 Department of Morphology, Surgery and Experimental Medicine and Laboratory for Technologies of Advanced Therapies (LTTA), University of Ferrara, Ferrara, Italy; 4 Mediapharma s.r.l., Via dei Vestini, Chieti, Italy

## Abstract

The ErbB tyrosine kinase receptor family has been shown to have an important role in tumorigenesis, and the expression of its receptor members is frequently deregulated in many types of solid tumors. Various drugs targeting these receptors have been approved for cancer treatment. Particularly, in breast cancer, anti-Her2/EGFR molecules represent the standard therapy for Her2-positive malignancies. However, in a number of cases, the tumor relapses or progresses thus suggesting that not all cancer cells have been targeted. One possibility is that a subset of cells capable of regenerating the tumor, such as cancer stem cells (CSCs), may not respond to these therapeutic agents. Accumulating evidences indicate that *miR-205-5p* is significantly downregulated in breast tumors compared with normal breast tissue and acts as a tumor suppressor directly targeting oncogenes such as Zeb1 and ErbB3. In this study, we report that *miR-205-5p* is highly expressed in BCSCs and represses directly ERBB2 and indirectly EGFR leading to resistance to targeted therapy. Furthermore, we show that *miR-205-5p* directly regulates the expression of p63 which is in turn involved in the EGFR expression suggesting a miR-205/p63/EGFR regulation.

Breast cancer is the most frequent type of cancer in women and despite the great improvement in diagnosis and treatment, relevant number of patients eventually relapses (SEER Cancer Statistics Review, 1975–2007, National Cancer Institute. Bethesda, MD, http://seer.cancer.gov/csr/1975_2007/, based on November 2009 SEER data submission, posted to the SEER web site, 2010). Recent studies have provided strong support for the cancer stem cell (CSC) hypothesis which holds that breast cancers are driven by a subpopulation of cells within the tumor which display stem cell properties.^1^ These properties include self-renewal, which generates other CSCs and differentiation, which generates populations of cells forming the bulk of the tumor. There is increasing evidence that CSCs are relatively quiescent cells, resistant to chemotherapy and radiation therapy and can therefore contribute to treatment resistance and relapse. It is therefore possible that relapses observed in ErbB2-positive breast cancer patients receiving adjuvant Trastuzumab (humanized antibody anti-Her2-Herceptin) or Lapatinib (small tyrosine kinases inhibitor molecule),^2, 3^ is due to the presence of CSCs that escape these therapeutic agents. Various mechanisms have been reported to cause resistance to targeted therapy, such as reduced ErbB2 expression, increased pro-survival signaling through alternative tyrosine kinases receptors or altered intracellular signaling leading to cellular over-proliferation.^4, 5^

Virtually all human genes are targeted by miRNAs,^6^ a class of non-coding endogenous small RNAs, which modulate the expression of their target genes through base pairing with the 3′ untranslated sequence (3′-UTR) of their target mRNAs.^7, 8^ MiRNA deregulation is widely described in cancer and has an important role in tumorigenesis.^9, 10^

*MiR-205-5p* is a highly conserved miRNA, expressed in stratified squamous epithelial-derived tissues^11^ and in mammary gland progenitor.^12^ It has been shown that *miR-205-5p* is downregulated in breast cancer and can specifically suppress ErbB3 expression.^13^ Moreover, *miR-205-5p* has been reported to mediate the epithelial to mesenchymal transition by targeting ZEB1 and ZEB2,^14, 15^ and it has a role in targeting several regulators of proliferation^16, 17^ suggesting its involvement in cellular differentiation, migration and proliferation. In addition, it has been reported that *miR-205-5p* is regulated by p63, a p53 family member resulting in epithelial to mesenchymal transition inhibition,^18^ whereas the loss of the p63/miR-205 axis enhances cell migration and metastasis in prostate cancer cells.^19^

The TP63 gene contains two promoters that produce two proteins: the full-length TAp63 that contains functional N-terminal transcriptional transactivation (TA) domains and the ΔNp63 protein, which lacks TA domains.^20^ p63 has central roles in epithelial development and despite the two isoforms share some common features,^20^ TAp63 mainly acts as tumor suppressor and ΔNp63 as an oncogene.^21, 22^

Here, we show that *miR-205-5p* is upregulated in patient-derived breast CSCs (BCSCs), compared with more differentiated tumor cells. More importantly, we show that *miR-205-5p* controls CSC phenotype targeting ErbB2, p63 and EGFR, contributing to targeted therapy resistance.

## Results

### BCSCs show low levels of ERBB2 and EGFR

**Figure 1:**
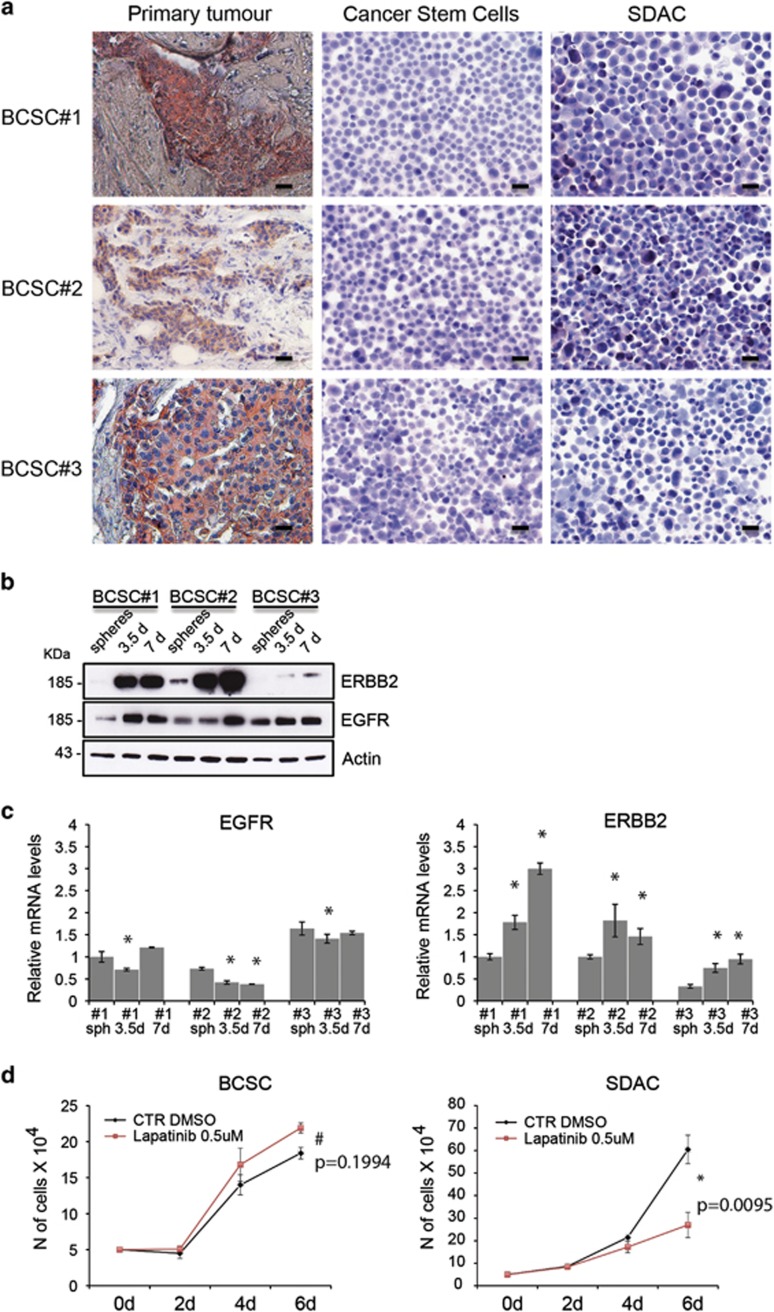
ERBB2 and EGFR expression pattern in BCSCs and during differentiation. (**a**) Immunohistochemistry of three different paraffin-embedded primary tumor tissues (left), CSCs lines derived from tumors (centre) and SDACs (right) stained with anti-HER2 antibodies (pink-brown) (Scale bar: 20 *μ*M). ERBB2 is expressed in primary tumors but not in CSCs lines and starts to be re-expressed under differentiating condition (SDAC). (**b**) Western blot analysis of BCSC lines (BCSC#1, BCSC#2 and BCSC#3) and stem cells under differentiating condition (SDAC) for 3.5 and 7 days using antibodies against ERBB2, EGFR and Actin as a loading control. The corresponding molecular weights are indicated on the left (KDa). ERBB2 and EGFR expression increases during differentiating condition. (**c**) qRT-PCR of ERBB2 and EGFR levels of BCSC lines (BCSC#1, BCSC#2 and BCSC#3) and SDAC of the same lines differentiated for 3.5 and 7 days. Data represent mean±S.D. of three different experiments analyzed in triplicate. Statistical significance was analyzed using Student's *T* test (*P<0.05). (**d**) BCSCs are resistant to Lapatinib treatment. Cell proliferation assay of BCSCs and SDAC untreated (ctr) or treated with 0.5 *μ*M of Lapatinib at the indicated time points (days). SDAC were differentiated for 3.5 days and then plated for growth curve analysis. BCSCs express low receptors levels and are more resistant to treatment than SDAC. Data represent mean±S.D. of three different experiments and *P* values are shown in the graphs

We characterized three patient-derived BCSC lines (BCSC #1, BCSC #2, BCSC #3) from three ErbB2-positive primary tumors. Immunohistochemistry analysis (Figure 1a) of the primary tumor confirms ErbB2 positivity in all three tumors, whereas CSCs derived from the tumors stain negative. Interestingly, when cells are grown as differentiating sphere-derived adherent cells (SDACs) for 14 days, they begin to show a faint positive staining. Western blot analysis confirms that spheroids retain very low expression levels of both ErbB2 and EGFR receptors that significantly increase when cells were grown as SDACs (Figure 1b). FACS analysis (Supplementary Figure S1) further confirms that indeed all three cell lines express variable (but generally low) levels of ErbB2 that again increase when cells are grown as SDACS. Interestingly, there is little correspondence between mRNA (Figure 1c) and protein levels for ErbB2 and almost none for EGFR, suggesting that expression changes in BCSCs and SDACs are at least in part mediated by non-transcriptional mechanisms. These data suggest that although CSCs show low expression levels of ErbB2 and EGFR, these receptors increase in tumor cells when they acquire a more differentiated phenotype.

### BCSCs are resistant to Lapatinib

We then tested the sensitivity of cell lines grown as mammospheres to Lapatinib, an ATP-competitive reversible small-molecule inhibitor of the ErbB2 and EGFR tyrosine kinases currently used in clinics as therapy for Her2-overexpressing metastatic breast cancers resistant to Trastuzumab-based regimens.^23^ As shown in Figure 1d, cells are resistant to treatment consistently with low expression levels of the receptors.

We then investigated whether cells regain sensitivity to this treatment when they are grown as SDAC and increase receptors expression. Indeed although BCSCs are completely resistant to treatment with Lapatinib (0.5 *μ*M), the same cells grown as SDACs appear significantly more sensitive (Figure 1d). These results suggest that failure of therapies to target BCSCs could be at least in part due to reduced expression of the receptors.

**Figure 2:**
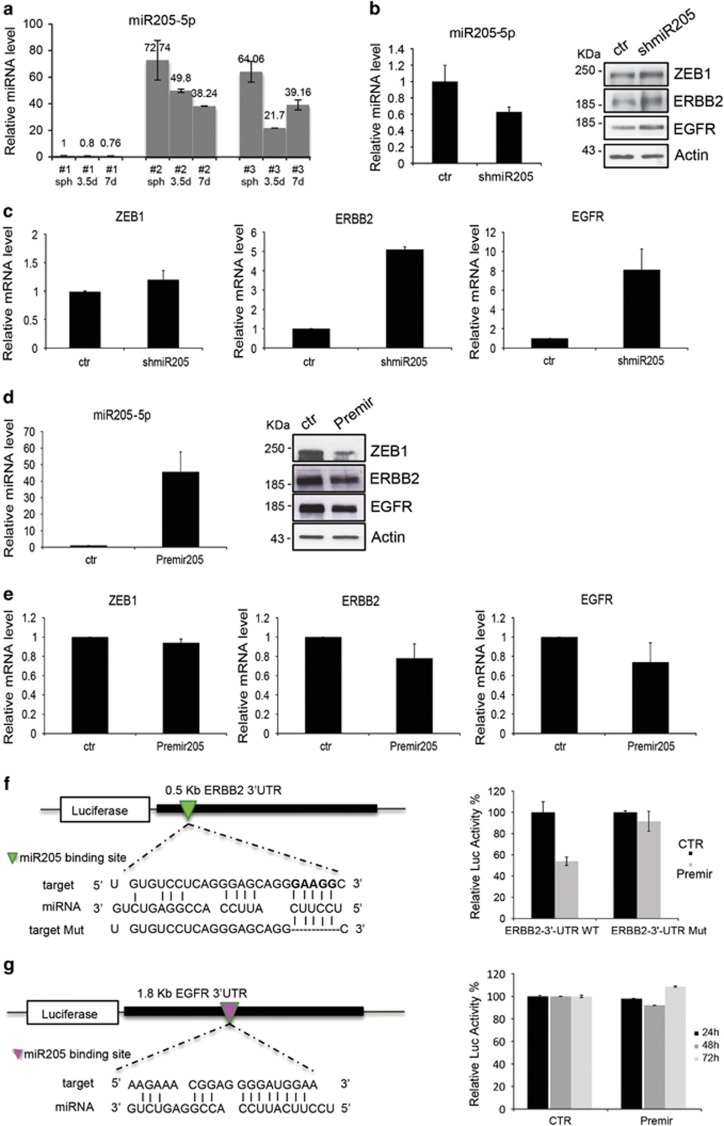
miR-205-5p regulates ERBB receptors expression in BCSCs. (**a**) qRT-PCR quantification of *miR-205-5p* expression in BCSC#1, BCSC#2 and BCSC#3 and SDAC differentiated for 3.5 and 7 days. *miR-205-5p* is downregulated during differentiation in all three stem cell lines tested. Data represent mean±S.D. of three different experiments analyzed in triplicate. (**b** and **c**) *miR-205-5p* regulates ERBB2 and EGFR expression. qRT-PCR and western blotting analysis of EGFR and ERBB2 expression levels in BCSC#1 infected with *miR-205-5p* silencing lentivector (shmiR-205-5p). miR-205 knockdown results in EGFR and ErbB2 upregulation both at the mRNA and protein levels. ZEB-1, a well-established miR-205 target, was used as a control to further confirm functional miR-205 silencing. (**d** and **e**) *miR-205-5p* overexpression results in ERBB receptors downregulation. qRT-PCR and western blot analysis of EGFR, ERBB2 and Zeb-1 expression levels in BCSC#1 infected with PremiR-205 lentivector. All qRT-PCR data represent mean±S.D. of three different experiments analyzed in triplicate. (**f**) *miR-205-5p* directly targets ERBB2 at 3'-UTR. Schematic model of the predicted ERBB2 3'-UTR binding site for *miR-205-5p* and alignment of the seed region with both wild-type and mutated ERBB2 3'-UTR (left). On the right, relative luciferase activity is shown. SKBR-3 cells were co-transfected for 24 h with pGL3-ERBB2 3'-UTR luciferase construct (WT or Mut 3'-UTR), premiR 205 construct or a control vector (CTR). The results represent mean±S.D. of three different experiments analyzed in triplicate. (**g**) EGFR is not a direct target of *miR-205-5p*. Representation of the interaction between *miR-205-5p* and the putative binding site on the wild-type EGFR 3'-UTR (left) and relative luciferase activity (right) in SKBR-3 cells transfected with premiR 205 construct or a control vector (CTR) at the indicated time points (hours)

There have been a number of studies describing a role of miRNA in the regulation of protein expression in normal and tumor breast stem cells. Moreover, a number of miRNAs have been shown to be involved during carcinogenesis,^24, 25^ and emerging evidence suggests that miRNAs also have essential roles in stem cell self-renewal and differentiation by negatively regulating the expression of key genes.^25^ Therefore, we performed a miRNA expression profile to identify miRNAs differentially expressed in BCSCs (BCSC #1) and SDACs collected at the indicated time point (7 days), and we identified 58 human miRNAs significantly differentially regulated (adjusted *P*<0.05). Among these, several have been previously shown to be involved in breast malignancy. Supplementary Figure S2 shows miRNAs that presented the highest differences between BCSCs and SDACs. We focused our attention on *miR-205-5p* because it has been previously reported^12, 26, 27, 28, 29^ to have a role in both normal mammary development and in breast cancer. MiR-205 has also been shown to be highly expressed in stem cell-enriched populations from normal mouse mammary gland, and thus may have a function also in maintaining the BCSC phenotype. We confirmed a higher expression of *miR-205-5p* in BCSCs as compared with SDACs in all three cell lines (Figure 2a). This inversely correlates with expression of ErbB2 and EGFR, thus suggesting that miR-205 is potentially a regulator of these receptors.

### *miR-205-5p* regulates ERBB2 and EGFR expression

To investigate whether *miR-205-5p* is indeed capable of regulating ErbB2 and EGFR expression, we silenced it in BCSC by cloning the *miR-205-5p* mature sequence in a pSIH-H1 shRNA expression lentivector. As shown in Figure 2b, *miR-205-5p* knockdown results in a significant EGFR and ErbB2 upregulation at protein levels as well as at mRNA levels (Figure 2c). ZEB-1, a well-established *miR-205-5p* target, was used as a control to confirm functional *miR-205-5p* silencing.

In addition, overexpression of *miR-205-5p* in BCSC #1 cells by infection with pCMV-RFP-2A-puro lentivector results in strongly reduced protein levels (Figure 2d) and in reduced mRNA levels (Figure 2e) of both ErbB2 and EGFR. Similar results were obtained using the same constructs in the other two BCSC lines (data not shown), suggesting a possible direct regulation of these two genes by *miR-205-5p*.

Because the main algorithms for miRNA target prediction fail to find the EGFR and ErbB2 as target of miR-205, we performed an *in silico* analysis using the RNAhybrid algoritm (http://bibiserv.techfak.uni-bielefeld.de/rnahybrid/submission.html) to identify putative target sites for *miR-205-5p* in the 3′-UTR of human ErbB2 (NM_004448) (Figure 2f) and of human EGFR (Figure 2g) (NM_005228). We found few putative binding region, therefore we cloned both ErbB2 and EGFR 3′-UTR containing the predicted *miR-205-5p* binding site into the pGL3 control vector downstream of the luciferase open reading frame. Co-transfection of mimic premiR-205 and the WT ErbB2 3′-UTR construct in SKBR3 cells results in a significant inhibition of the luciferase activity compared with cells in which the WT ErbB2 3′-UTR construct was co-transfected with a control vector (Figure 2f). Mutation of *miR-205-5p* binding site within the ErbB2 3′-UTR (ERBB2-3′-UTR Mut) abolishes the ability of *miR-205-5p* to regulate the luciferase expression resulting in the increase of luciferase activity (Figure 2f). In contrast, different results were obtained co-transfecting *miR-205-5p* and the WT EGFR 3′-UTR construct in SKBR3 cells. Indeed, as shown in Figure 2g, *miR-205-5p* was not able to decrease luciferase activity, even at longer time points (24, 48 and 72 h after transfection). These data suggest that *miR-205-5p* might modulate EGFR expression indirectly, by targeting others key factors involved in EGFR regulation.

### *miR-205-5p* controls p63 expression

To further investigate the indirect regulation of EGFR expression by *miR-205-5p,* we studied the role of p63 in this pathway, because it has been reported to promote the transcription of EGFR.^30^ Interestingly, it has also been shown that p63 controls *miR-205-5p* expression.^18, 19^

**Figure 3:**
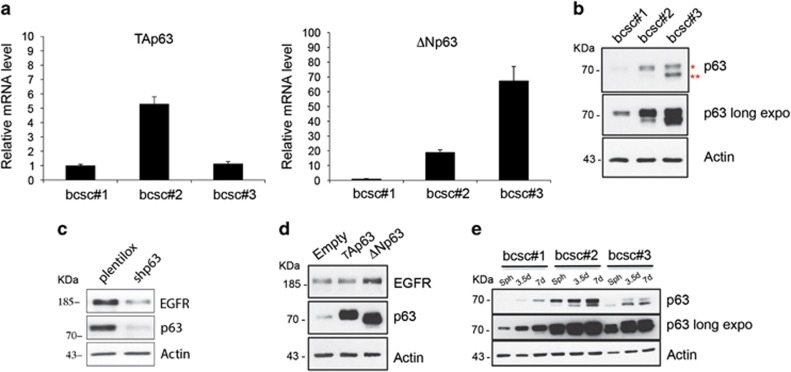
p63 expression pattern in BCSCs and during differentiation. (**a**) p63 expression pattern in BCSCs. TAp63 (left) and ΔNp63 (right) expression levels in BCSC#1, BCSC#2 and BCSC#3 were assessed by qRT-PCR. All three stem cell lines tested show p63 detectable levels. (**b**) Western blot analysis of p63 protein levels in BCSC#1, BCSC#2 and BCSC#3 normalized with Actin levels. Numbers on the left indicate molecular weight (KDa) and symbol on the right indicate * Tap63 and ** ΔNp63, respectively. (**c**) EGFR protein levels in BCSC#1 infected with p63 silencing (shp63) lentivector. p63 silencing leads to EGFR downregulation. (**d**) ΔNp63 and not Tap63 regulates EGFR expression. Western blot of EGFR protein levels in BCSC#1 infected with TAp63 or ΔNp63 overexpression lentivector or a control vector (Empty). (**e**) p63 expression pattern in BCSC#1, BCSC#2 and BCSC#3 and SDAC collected at 3.5 and 7 differentiation days. Western blot of p63 levels shows an increase of both TAp63 and ΔNp63 isoforms during differentiating condition (middle: long exposure to better evaluate p63 expression in all three BCSC lines)

**Figure 4:**
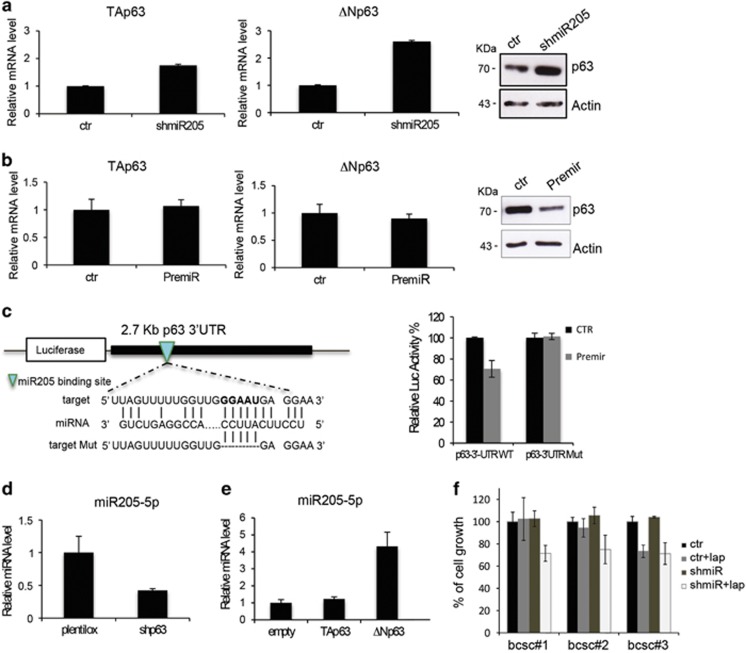
*miR-205-5p* regulates p63 expression in BCSCs. (**a**) *miR-205-5p* regulates p63 levels. qRT-PCR (left) and western blotting analysis (right) of p63 expression levels in BCSC#1 infected with *miR-205-5p* silencing lentivector (shmiR-205-5p). (**b**) qRT-PCR (left) and western blotting (right) of p63 expression levels in BCSC#1 infected with Premir-205 lentivector. *miR-205-5p* overexpression results in p63 downregulation mainly at protein levels. (**c**) *miR-205-5p* directly targets p63-3'-UTR. On the left, putative *miR-205-5p* binding site on p63 wild-type 3'-UTR and alignment of the seed sequence with both WT and mutated p63 3'-UTR. On the right, SKBR-3 cells were co-transfected for 48 h with pGL3-p63 3'-UTR luciferase construct (WT or Mut 3'-UTR), premiR 205 construct or a control vector (CTR). Cloning p63 3'-UTR WT, and not the mutated one, into a luciferase reporter gene leads to diminished luciferase activity in the presence of Premir-205. (**d**) p63 regulates *miR-205-5p* expression. qRT-PCR of *miR-205-5p* expression levels in BCSC#1 infected with shp63 lentivector (shp63) or a control vector (plentilox). (**e**) qRT-PCR of *miR-205-5p* expression levels upon Tap63 or ΔNp63 overexpression in BCSC#1. ΔNp63 overexpression results in *miR-205-5p* upregulation. (**f**) *miR-205-5p* downregulation re-sensitize BCSCs to Lapatinib treatment. Percentage of cell growth of BCSC#1, BCSC#2 and BCSC#3 infected with shmiR-205-5p lentivector (shmiR-205-5p) or a control vector (CTR) and treated or untreated with 0.5 *μ*M Lapatinib for 6 days

To evaluate the potential role of p63 in *miR-205-5p* -ERBB family axis, we first checked the expression levels of p63 in our model. As shown in Figure 3, all three lines tested express detectable mRNA (Figure 3a) and protein (Figure 3b) levels of p63. In addition, we tested whether p63 was also able to drive the expression of EGFR in BCSCs as previously reported. As shown in Figure 3c, silencing of p63 in BCSC #1 cells results in reduced expression of EGFR, whereas upregulation of the ΔN but not the TA isoform results in increased EGFR levels (Figure 3d). Interestingly, p63 levels increase in more differentiated cancer cells as compared with cells grown as spheroids in parallel with the reduction of *miR-205-5p* expression (Figure 3e). We therefore investigated whether *miR-205-5p* is capable of regulating p63 expression. As shown in Figure 4a, *miR-205-5p* knockdown results in a significant p63 upregulation both at the mRNA and protein levels. Consistently, miR-205-5p overexpression results in reduced p63 levels (Figure 4b). Bio-informatic analysis of p63 3′-UTR identified a putative *miR-205-5p* seed region (Figure 4c, left). We therefore cloned the p63 3'-UTR containing the *miR-205-5p* binding site into the pGL3 control vector downstream the luciferase gene. Co-transfection of premiR-205 and the WT p63 3′-UTR construct in SKBR3 cells resulted in significant inhibition of luciferase activity compared with the cells in which the WT p63 3′-UTR construct was co-transfected with a control vector (Figure 4c, right). These data were confirmed by mutation of *miR-205-5p* binding site within the p63 3′-UTR (p63-3'-UTR Mut) that abolishes the ability of *miR-205-5p* to regulate the luciferase expression leading to an increase of luciferase activity. We therefore believe that *miR-205-5p* regulates expression of EGFR through regulation of ΔNp63. Interestingly, we confirm that as reported in literature, p63 regulates *miR-205-5p* expression thus creating a regulatory feedback loop. In fact, p63 silencing results in reduced *miR-205-5p* levels (Figure 4d), whereas upregulation of the ΔN but not the TA isoform results in significant increase of *miR-205-5p* levels (Figure 4e).

We therefore sought out to re-sensitize BCSCs to Lapatinib treatment downregulating *mir-205-5p*, that we showed is responsible for reduced expression of EGFR and ErbB2 in these cells. As shown in Figure 4f, silencing *miR-205-5p* in BCSCs and treating them with Lapatinib strongly reduces cell proliferation, sensitizing cells to anti-Her2/EGFR treatments. These findings indicate that *miR-205-5p* is able to regulate ErbB receptors expression thus leading to targeted therapy resistance of BCSCs.

## Discussion

Growing evidence suggest that a subset of cells within the tumor, refered as CSCs are capable of escaping anti-cancer treatment driving tumor progression, metastasis and relapse.^1^ Many studies have therefore focused on the identification of pathways that are essential in determining the CSC phenotype. It is well known that miRNAs contribute to carcinogenesis and tumor development acting as oncogenes or as tumor suppressors depending on specific targets and tumor microenvironment. Altered *miR-205-5p* expression has been involved in several types of solid tumors and, to date, its target include tumor suppressors like PTEN^12^ and SHIP2,^31^ oncogenes such as HER3,^32^ PKCɛ,^17^ pro-metastatic factors Zeb1 and Zeb-2,^14^ and the angiogenic gene VEGFA.^32^ Moreover, *miR-205-5p* has been shown to be essential for mouse development,^33, 11^ particularly for the expansion of progenitor and stem cell populations in epidermis, hair follicles and more importantly in mammary gland during neonatal development;^33, 12^ therefore, we hypothesize its mis-regulation could be translated in maintaining the CSC phenotype. In breast cancer, *miR-205-5p* was found to be either up- or downregulated compared with normal tissue, but its expression in BCSCs remains still unknown. Here, we show that *miR-205-5p* is highly expressed in human BCSCs compared with more differentiated tumor cells and that it directly targets ERBB receptors leading to their downregulation. In fact while it was known that ERBB3 is a direct target of *miR-205-5p,* we show that it also regulates ErbB2 and EGFR. While ErbB2 appears to be a direct target of this miR, EGFR regulation is mediated through the regulation of p63 that has already been shown to be able of regulating transcription of EGFR.^30^ Indeed, here we show for the first time that p63 is a direct target of *miR-205-5p* and confirm previous reports showing that in turn ΔNp63 regulates *miR-205-5p* expression.^19, 18^ These data show that, therefore, there is a feedback loop finely regulating expression of *miR-205-5p* and p63 that have a role in determining some of the phenotypic features of BCSCs including surface expression of ERBB receptors.

Intriguingly, we show that low expression of ERBB receptor family members in BCSCs contributes to resistance of these cells to agents such as Lapatinib used in breast cancer therapy. Survival of these cells could then lead to tumor progression and suggests that *miR-205-5p* could be an important target to improve outcome of patients with Her2-overexpressing breast cancer.

In conclusion, we want to point out that we identified for the first time two new direct targets of *miR-205-5p* and shown that this miRNA has an important role in determining BCSCs phenotype and contributes to their resistance to targeted therapy.

## Materials and Methods

### BCSC isolation and culture

BCSCs were isolated from human breast cancer tissues obtained from patients as previously described^34^ and were cultured in a selective medium^34^ supplemented with 10 ng/ml βFGF (Peprotech, London, UK), 20 ng/ml EGF (Peprotech) to a final concentration 5 × 10^4^/ml in ultra low attachment flask (Corning, New York, NY, USA) at 37 °C in a 5% (v/v) CO_2_ humidified chamber. BCSCs were induced to differentiate in order to obtain SDACs by culturing them in adherent condition in D-MEM with high glucose (Euroclone, Milan, Italy) supplemented with 10% (v/v) fetal bovine serum (Euroclone). The tumor s were histopathologically classified as follows: BCSC#1 is an invasive ductal carcinoma, grading G2, ER 90%, PR 60%, HER2/neu 3+ and ki67 >10% BCSC#2 is an invasive ductal carcinoma, grading G2, ER 90%, PR 60%, HER2/neu 3+ and ki67 25%, BCSC#3 is an invasive ductal carcinoma, grading G2, ER 80%, PR 80%, HER2/neu 3+ and ki67 >10%. HER2 status has been assigned according to the FDA guidelines.

### Constructs

To generate *miR-205-5p* expression lentiviral vector, a 326 bp fragment carrying pre-miR-205 was amplified from H1299 genomic DNA by the Platinum Taq polymerase high fidelity (Life Technologies, Waltham, MA, USA) using PCR primers: miR-205 F 5′-cggctagccgaggtccttgacatct-3′ and miR-205 R 5′-ccctcgagggcctaagtcagagtta-3′.

The amplified fragment was cloned into pLenti-CMV-RFP-2A-Puro (Applied Biologicals Marerials Inc., Richmond, BC, Canada) lentiviral vector at *Nhe*-*Xho*I sites.

To generate shRNA miR-205 lenti-vector, we cloned *miR-205-5p* into pSIH-H1-copGFP Vector (System Biosciences, Mountain View, CA, USA), at *Eco*RI-*Bam*HI sites, using PCR oligonucleotides:

shmiR-205 1 5′-gatcctccttcattccaccggagtctgcttcctgtcagacagactccggtggaatgaaggatttttg-3′ shmiR-205 2 5′-aattcaaaaatccttcattccaccggagtctgtctgacaggaagcagactccggtggaatgaaggag-3′.

To generate a luciferase reporter carrying the ErbB2-3'-UTR with a putative *miR-205-5p* binding site, we amplified a 512 bp fragment by PCR from the first nucleotide after the stop codon to the last nucleotide before the polyadenylation signal from human genomic DNA using the following primers: ERBB2-UTR SpeF 5′-gactagtcaccagaaggccaagtccg-3′ and ERBB2-UTR SpeR 5′-ggactagtcctcatctttaaaaaaacaaaac-3′. The fragment, after *Spe*I restriction, was ligated to a compatible *Xba*I linearized pGL3 Control vector (Promega, Madison, WI, USA). The *miR-205-5p* predicted target site (5 bp, GAAGG) was deleted by PCR using the following primers: Her2MutFw 5′- gccctgatgtgtcctcagggagcaggcc-3′ and Her2MutRev 5′-tgatgccagcagaagtcaggcctgctcc-3′ with QuikChange Lightning Site-Directed Mutagenesis kit (Agilent Technologies, Santa Clara, CA, USA).

The same procedure was used to clone 2770 bp p63 3'-UTR fragment in a pGL3 Control vector using the following primers: p63UTR-SpeIF 5'-ggccactagtgcctcaccatgtgagctcttc-3' p63UTR-SpeIR 5′-ggccactagtgcatgtcctggcaaacaaaaagag-3′ as previously described.^35^ Mutation (5 bp, GGAAU) was introduced into the miRNA binding site with QuikChange Lightning Site-Directed Mutagenesis kit (Agilent Technologies) using the following primers: Fwp63Mut gtttttggttggaggaaaattcttaaaaggcccatagcagc

Revp63Mut gctgctatgggccttttaagaattttcctccaaccaaaaac.

EGFR 3'-UTR WT luciferase reporter vector was kindly provided by CM Croce (Ohio State University Wexner Medical Center and Comprehensive Cancer Center, Columbus, OH, USA).

To generate TAp63α-Tween and ΔNp63α-Tween expression lentiviral vectors, we subcloned inserts from TAp63α−pcDNA and ΔNp63α−pcDNA constructs, kindly provided by Prof. G Melino,^36^ into *Xba*I-*Xho*I unique sites of Tween lentiviral vector^37^ under the control of hCMV promoter. This vector constitutively expresses GFP under the control of hPGK promoter.

For shp63 construct, to downregulate p63 expression, the pLL3.7-p63-7.2 was generated by insertion in pLL3.7 vector (Addgene plasmid 11795, Addgene, Cambridge, MA, USA) of oligos targeting the following sequence: GAGTGGAATGACTTCAACTTT.^38^

The cloning was performed according to the pLL3.7 protocol from the Tyler Jacks laboratory at MIT (https://www.addgene.org/static/data/94/67/16242780-af64-11e0-90fe-003048dd6500.pdf).

A CMV-EGFP reporter cassette is included in the vector to monitor expression.

All PCR products were verified by DNA sequencing.

### Infections

Lentiviruses were produced by transient cotransfection of a three-plasmid expression system in the packaging 293T cells, using the calcium phosphate transfection kit (Invitrogen, Life Technologies). Cells were incubated for 7 h with the transfection reagents and viral supernatant was collected 48 h after transfection and filtered through 0.45 μm pore vacuum sterile filtration system (Millipore, Life Science, Darmstadt, Germany). Then, BCSCs were plated in a six-well ultra-low attachment plate (Corning) with viral supernatant and 4 μg/ml of polybrene. Plates were centrifuged for 45 min at 1800 r.p.m. and incubated at 37 °C for 75 min in a 5% CO_2_ humified chamber. Cells were then washed twice and replated in fresh medium.^39^ Infection efficiency was assessed by flow cytometry (FACSCanto II Instrument, BD Biosciences, San Jose, CA, USA) 48 h post-infection evaluating the percentage of GFP-positive or RFP-positive cells measured. Data were analyzed with CELLQuest software (BD Biosciences).

### Quantitative reverse transcription-PCR

For miRNA detection, RNA was extracted from BCSCs by using miRVana miRNA Isolation kit (Ambion by Life Technologies). A total of 50 ng of RNA was used for reverse transcription using TaqMan MicroRNA Reverse Transcription Kit (Applied Biosystem by Life Technologies) with the following stem loop specific primers: *miR-205-5p* RT: 5′-gttggctctggtgcagggtccgaggtattcgcaccagagccaaccagact-3′ and U44-RT: 5′-gttggctctggtgcagggtccgaggtattcgcaccagagccaacagtcagtt-3′.

Real-time PCR was performed by using FastStart Universal Probe Master (Rox) (Roche, Basel, Switzerland) and Universal Probe Library, Probe #21 (Roche) using the following primers: *miR-205-5p* Fw 5′-gcggcggtgtagtgtttccta-3′ and universal Reverse primer: 5′-gtgcagggtccgaggt-3′. *miR-205-5p* expression was calculated relative to U44 rRNA with the following primer: 5′-gcggcggcctggatgatgatag-3′ with an amplification protocol as follows: one cycle of 95 °C for 10 min and 40 cycles of 95 °C for 15 s and 60 °C for 1 min on an Applied Biosystem 7900HT Sequence Detection System (Applied Biosystems, Waltham, MA, USA). Relative quantification of miRNA expression was calculated according to the comparative method of 2-^ΔΔCT^.

For detection of other genes, a total of 500 ng of RNA was used for reverse transcription using TaqMan Reverse Transcription Reagents (Applied Bioisystem, Life Technologies) and Real-time PCR was performed by using the Platinum SYBR Green qPCR SuperMix UDG with Rox (Invitrogen, Life Technologies), with an amplification program as follows: one cycle of 95 °C for 3 min and 40 cycles of 95 °C for 20 s and 60 °C for 1 min. The reaction was followed by a melting curve protocol according to the specification of the ABI 7900HT instrument (Applied Biosystems). Primers used were as follows: Tap63 (NM_003722.4) F: 5′-ttgagattagcatggactgtatcc-3′and R: 5′-gttctgaatctgctggtccat-3′ ΔNp63 (NM_001114980.1) F: 5′-ggttggcaaaatcctggag-3′ and R: 5′-ggttcgtgtactgtggctca-3′. For ErbB2 (NM_004448.2) F: 5′-gggaaacctggaactcacct-3′ and R: 5′-ccctgcacctcctggata-3′ for EGFR (NM_005228.3) F: 5′-ttcctcccagtgcctgaa-3′ and R: 5′-gggttcagaggctgattgtg-3′ for ZEB1 F: 5′-gcacaaccaagtgcagaaga-3′ and R: 5′-gcctggttcaggagaagatg-3′.

All genes expression were normalized using human β-actin as housekeeping gene, and primers used were ActF 5′-cagctcaccatggatgatgatatc-3′ and ActR 5′-aagccggccttgcacat -3′. Relative quantification of gene expression was calculated according to the comparative method of 2-^ΔΔCT^.

### Microarray

Total RNA was extracted from BCSC #1 BCSCs and from BCSC #1 differentiated cells at 7 days, according to Trizol protocol (Ambion by Life Technologies). Total RNA was used for miRNA microarray analysis (G4470B, Agilent Technologies). This chip allows the simultaneous analysis of 723 human miRNAs (miRBase release 10.1). RNA labeling and hybridization were performed in accordance to the manufacturer's indications. Agilent scanner and the Feature Extraction 10.5 software (Agilent Technologies) were used to obtain the microarray raw data. Microarray results were analyzed using the GeneSpring GX 12 software (Agilent Technologies). Data transformation was applied to set all the negative raw values at 1.0, followed by Quantile normalization and log2 transformation. Differentially expressed miRNAs were identified by using a moderated *t*-test and Benjamini-Hochberg correction (adjusted *P*<0.05). Differentially expressed genes were used in Cluster Analysis, using the Pearson correlation as a measure of similarity.

### Western blotting

Proteins were extracted with a lysis buffer (TRIS-HCl 50 mM pH 8, NaCl 150 mM, Triton X-100 1%, NaF 100 mM, EDTA 1 mM, MgCl_2_ 1 mM, Glycerol 10%) containing a protease inhibitor cocktail (Sigma-Aldrich, St. Louis, MO, USA) and a phosphatase inhibitor cocktail (Roche) as previously described.^40^ Equal amounts of total protein were subjected to SDS-PAGE and then electrotransferred to nitrocellulose membranes. The membranes were blocked with 5% non-fat dry milk in PBS with 0,1% Tween 20 and incubated overnight using the following antibodies: anti β-Actin A5441 (Sigma-Aldrich), anti-EGF Receptor (D38B1) XP Rabbit mAb (Cell Signaling, Danvers, MA, USA), anti-ErbB2 (D8F12) XP Rabbit mAb (Cell Signaling) anti-ZEB1 (Millipore, Life Science), anti-p63 Y4A3 (Sigma-Aldrich) or anti-p63 alpha (D2K8X) XP Rabbit mAb (Cell Signaling). After wash, membranes were hybridized with horseradish peroxidase-conjugated secondary antibodies (rabbit and mouse, Bio-Rad, Hercules, CA, USA). Detection was performed with Plus-ECL chemiluminescence kit (PerkinElmer, Inc., Waltham, MA, USA) or with SuperSignal West Dura extended duration substrate kit (Thermo Scientific, Waltham, MA, USA).

### Immunohistochemistry

BCSCs and SDACs derived from BCSC#1, BCSC#2 and BCSC#3 lines, were spotted on microscope slides with cytospin at 900 r.p.m. for 3 min and then were fixed in formalin 10% neutral buffered for 15 min. BCSC, SDAC and paraffin-embedded primary tumor tissues were stained using rabbit polyclonal (HercepTest, Dako, Glostrup, Denmark) antibodies following the manufacturer's instructions. Antigen-antibody reaction was visualized using an anti-rabbit #K4003 polymer-based detection system (EnVision Kit, Dako), and using diaminobenzidine as the chromogen. In control sections, the specific primary antibody was replaced with rabbit non immune serum. Primary tumors BCSC #1, BCSC#2 and BCSC#3 were scored according to HercepTest (HT) (Dako) and classified as HT 3+.^41^

### Luciferase assay

Human breast carcinoma cell line (SKB-R3) was grown in McCoy's 5A Medium (Euroclone, Milan, Italy) supplemented with 10% (v/v) fetal bovine serum (Euroclone). A total of 8 × 10^4^ SKB-R3 cells were seeded in 12-well dishes 24 h before transfection. Three hundred and twenty-five nanograms of pGL3 vectors, 650 ng of pLenti-CMV-RFP-2 A-Puro vectors and 10 ng of Renilla luciferase vector were co-transfected using Lipofectamine 2000 (Invitrogen by Life Technologies). Luciferase activities of cellular extracts were measured 24 and 48 h after transfection for ErbB2 and p63 3'-UTR, respectively, and for 24, 48 and 72 h after transfection for EGFR 3'-UTR, by using a Dual-Glo-Luciferase Reporter Assay System (Promega, Fitchburg, WI, USA) with a Lumat LB 9507 luminometer. Efficiency of transfection was normalized using Renilla luciferase activity.

### FACS analysis

For flow cytometric analysis of EGFR and ErbB2 surface markers, 20 × 10^4^ cells per sample, for all three BCSCs and SDACs tested (BCSC#1, BCSC#2 and BCSC#3), were washed in PBS, resuspended in 100 *μ*l of specific antibody diluted in 0.5% BSA, and incubated for 20 min at room temperature. For EGFR and ErbB2 staining 10 *μ*g/ml Trastuzumab (Roche) and Cetuximab (Merck Serono, Darmstadt, Germany) were used, respectively. Then, samples were incubated for 20 min in the dark with secondary fluorescent anti-human-R-PE-conjugated antibody (H10104, Life Technologies, 1 : 300 in 0.5% BSA). Cell viability solution (555815, BD Biosciences) was used for detection of non-viable cells according to the manufacturer's protocol. Samples were then washed and stored at 4 °C in the dark until acquisition.

A FACSCantoII flow cytometer, running with FACSDiVa software (BD Biosciences), was used for sample acquisition and analysis.

### Cell proliferation assay

BCSCs, SDACs and BCSCs infected with shmiR-205-5p were seeded into six-well plate at 5 × 10^4^ cells per well. Viable cell count was performed with Trypan Blue reagent (Sigma-Aldrich) at the indicated time points. When indicated, cells were treated with 0.5 *μ*M Lapatinib (Biovision, Milpitas, CA, USA).

### Bioinformatics

*miR-205-5p* target sites on p63 3'-UTR, ERBB2 3'-UTR and EGFR 3'-UTR were predicted by RNA Hybrid software available at http://bibiserv.techfak.uni-bielefeld.de/rnahybrid/submission.html

## Abbreviations

BCSC: breast cancer stem cells
CSC: cancer stem cells
HT: hercept test
SDAC: sphere-derived adherent cells
