# Supplementary figures and images for: miR-205-5p-mediated downregulation of ErbB/HER receptors in breast cancer stem cells results in targeted therapy resistance
Source: Cell Death Dis. 2015 Jul 16;6(7):e1823–. doi: 10.1038/cddis.2015.192 (PMC4650737; doi:10.1038/cddis.2015.192)

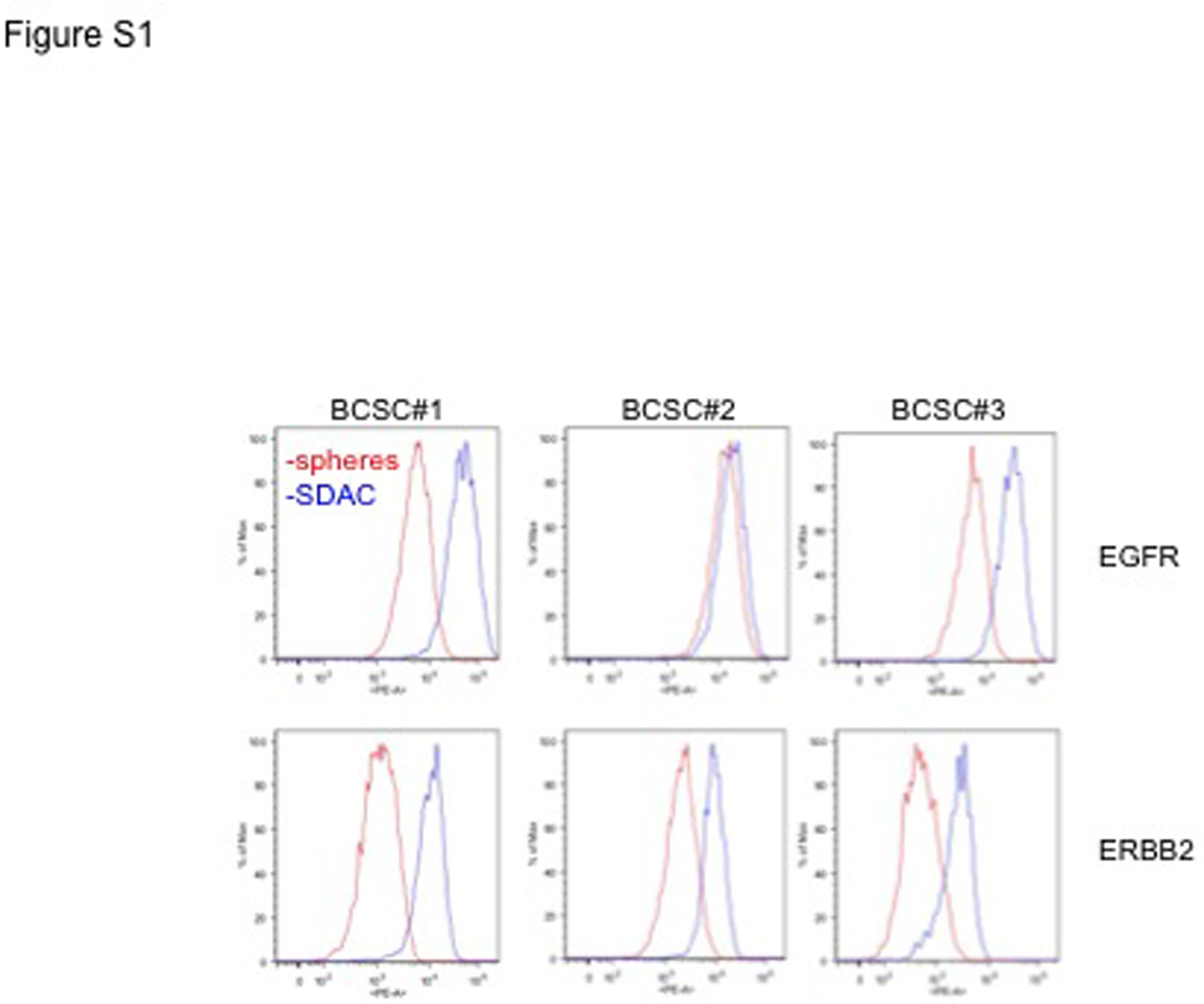

Supplement: Supplementary Figure 1 [file cddis2015192x1.tif]

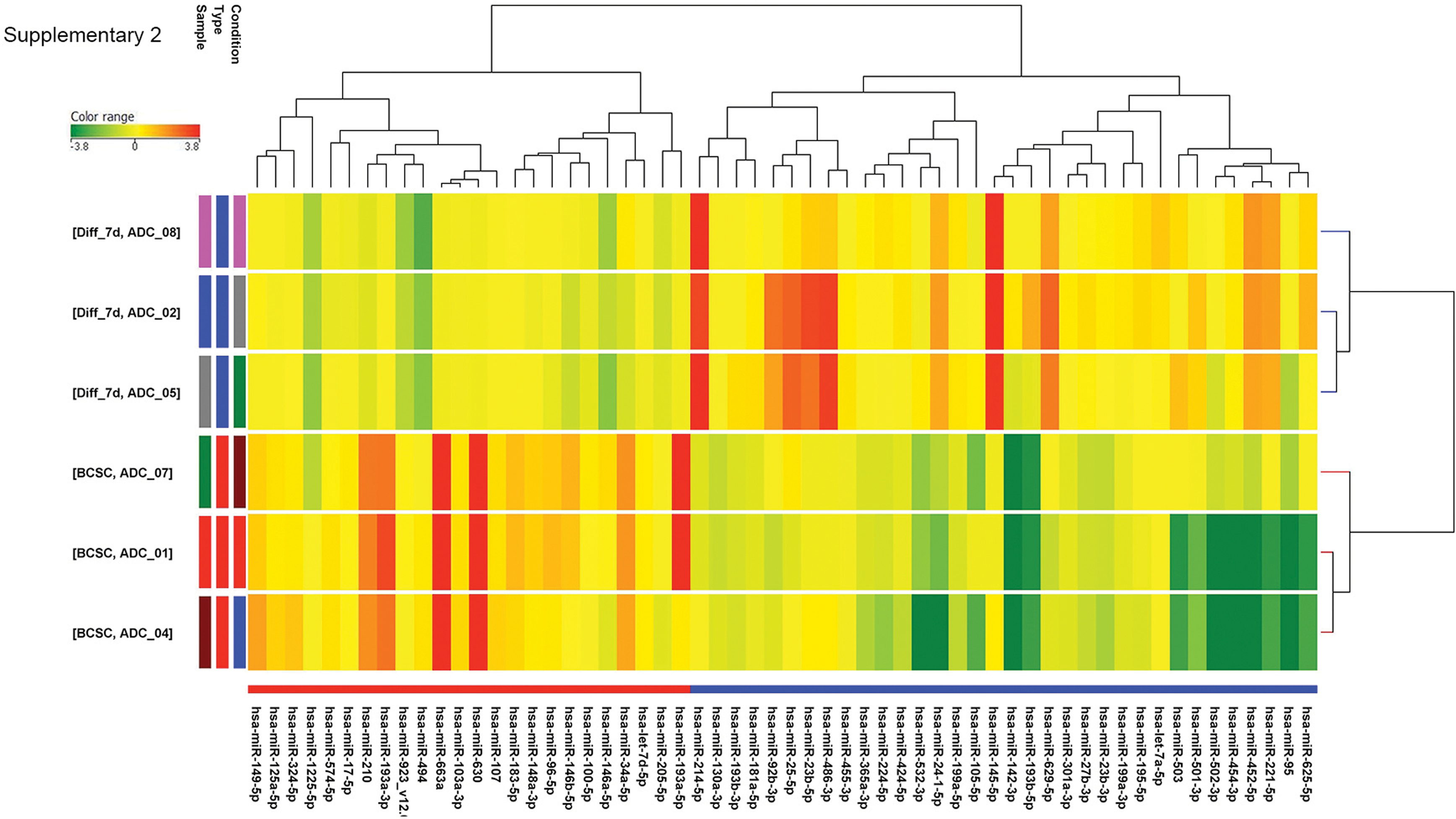

Supplement: Supplementary Figure 2 [file cddis2015192x2.tif]
