# Supplementary material for: miR-205-5p-mediated downregulation of ErbB/HER receptors in breast cancer stem cells results in targeted therapy resistance
Source: Cell Death Dis. 2015 Jul 16;6(7):e1823–. doi: 10.1038/cddis.2015.192 (PMC4650737; doi:10.1038/cddis.2015.192)
Supplement: Supplementary Figure Legends [file cddis2015192x3.docx]

**Figure S1**

FACS analysis of the same BCSCs lines grown as spheres (red) or SDAC differentiated for 3,5 days (blue) and stained with anti EGFR and ERBB2 antibodies.

**Figure S2**

Cluster analysis of BCSCs and SDAC after 7 days of differentiation performed using 58 human miRNAs differentially expressed in BCSCs and SDAC (adjusted p < 0.05). *miR-205-5p* is over-expressed in BCSC and down-regulated in SDAC.
